# The Efficacy of Platelet-Rich Plasma Augmentation in Microfracture Surgery Osteochondral Lesions of the Talus: A Systematic Review and Meta-Analysis

**DOI:** 10.3390/jcm12154998

**Published:** 2023-07-29

**Authors:** Inha Woo, Jeong Jin Park, Hyun-Gyu Seok

**Affiliations:** Department of Orthopedic Surgery, Yeungnam University Medical Center, Daegu 42415, Republic of Korea; buonggiorno39@gmail.com (I.W.); wjdwls3912@ynu.ac.kr (J.J.P.)

**Keywords:** platelet-rich plasma, microfracture, osteochondral lesion of the talus, meta-analysis

## Abstract

The appropriate surgical management of osteochondral lesions of the talus (OLT) remains a challenge for foot and ankle surgeons. Currently, microfracture (MF) is the first-line operative treatment for small osteochondral lesions. However, the fibrous cartilage regenerated after MF is mechanically inferior to hyaline cartilage regeneration and is susceptible to deterioration over time. Thus, this meta-analysis aimed to elucidate the efficacy of platelet-rich plasma (PRP) augmentation compared with MF only or other adjuvant supplementations combined with the PRP + MF group (others) for the management of OLT. We searched the PubMed, Embase, Web of Science, and Cochrane Library databases for studies that compared the clinical outcomes of patients who underwent MF only and those who underwent PRP or other adjuvant materials such as hyaluronic acid or BST-CarGel. After the screening, four randomized controlled trials and one quasi-randomized controlled trial were included in this review. We used the following tools for clinical evaluation: the American Orthopedic Foot and Ankle Society (AOFAS) score, Ankle–Hindfoot Scale score, Visual Analog Scale (VAS) score for pain, and the Foot and Ankle Ability Measure (FAAM) score. The standardized mean difference (SMD) was used to analyze the differences in outcomes between groups. Patients in the PRP + MF group had superior final VAS and AOFAS scores to the MF only group. (both *p* < 0.01) However, no significant improvements between baseline and final follow-up were noted in either score. In addition, there was no remarkable difference in the overall FAAM pain measures between the two groups. The PRP + MF and others groups revealed no significant effect differences in the clinical scores. The results of this analysis suggest that PRP + MF would be more favorable and effective than MF only or additional adjuvant supplementation.

## 1. Introduction

Osteochondral lesions of the talus (OLT) are defined as the detachment of articular cartilage fragments with or without subchondral bone. A traumatic event is considered a predisposing factor of OLT due to the high incidence in individuals with ankle sprains and among athletes [1,2,3,4,5]. However, the etiology of the lesions has not yet been fully elucidated and there are various contributing factors, such as certain vascular conditions, infection, hormonal disturbance, and ossification disorders [6,7].

In addition to clarifying the etiology, the management of OLT remains a challenge for foot and ankle surgeons [8]. The inappropriate management of OLT may cause osteoarthritis of the ankle joint, leading to devastating outcomes that require joint-sacrificing surgeries [9]. Therefore, early detection and proper management are important [10]. Various surgical management options including debridement, microfracture (MF), osteochondral autograft transplantation, and autologous chondrocyte implantation have been utilized in previous studies [4,11]. Of these, arthroscopic MF is the most widely used method in the treatment of OLT due to the simplicity of the technique [12]. MF has shown excellent short-term functional outcomes in small-sized lesions [12,13]. However, concerns over the efficacy of cartilage treatment in large lesions and poor-quality fibrocartilage regeneration remain [12,14].

Recently, the addition of biological compounds during surgery improved the clinical outcomes in patients with cartilage-injury diseases such as osteoarthritis and osteochondral lesions [15]. Platelet-rich plasma (PRP), one of these compounds, contains a high concentration of platelets that encompass and release numerous growth factors [16,17]. These growth factors are effective in tendon and diabetic wound healing and in the repair of cartilage defects [18,19,20,21,22]. In particular, PRP as an adjunct to MF for knee osteochondral lesions or osteoarthritis (OA) has had positive clinical outcomes in some studies [23,24,25]. The rationale for PRP use is to provide high physiological doses of growth factors to the defected chondral lesions, enhancing chondrogenesis and inducing the chemotaxis of mesenchymal stem cells (MSCs) [26,27] However, inconsistent study findings have been reported in the treatment of ankle OLT [6,28,29,30,31]. To our knowledge, there is a paucity of systemic reviews investigating the role of PRP in augmenting MF [32]. Additionally, we tried to investigate the efficacy of adjuvant materials such as hyaluronic acid combined with PRP and MF. As such, we conducted a meta-analysis to elucidate the efficacy of PRP in MF surgery for the management of OLT. We believe that this study will contribute to the literature by updating the existing knowledge and synthesizing new information on the efficacy of PRP as an adjunct to arthroscopic MF for the management of OLT.

## 2. Materials and Methods

This meta-analysis was performed in accordance with the Preferred Reporting Items for Systematic Reviews and Meta-Analysis (PRISMA) guidelines [33].

### 2.1. Search Strategy and Study Selection

Relevant studies published between 1 January 2003 and 20 May 2023 were systematically searched in PubMed, Embase, Web of Science, and the Cochrane Library. The search terms included free-text terms and Medical Subject Heading (MeSH) terms related to our desired topics: a combination of synonyms (‘osteochondral lesions’ OR ‘OLT’) AND (‘talus’ OR ‘talar’) AND (‘platelet-rich plasma’ OR ‘PRP’). In addition, the reference lists of previously published articles were manually searched for additional eligible studies.

The articles were selected based on the following inclusion criteria: (1) studies that compared the clinical outcomes of patients who underwent PRP as an adjunct to arthroscopic MF and those who underwent other procedures such as arthroscopic MF only or the injection of supplements such as hyaluronic acid after arthroscopic MF; (2) studies that were designed as randomized or quasi-randomized controlled trials; (3) studies that included a control or comparison group; (4) studies with a level of evidence ≥ 2. The exclusion criteria were as follows: (1) reviews, case reports, or other indistinct forms; (2) substudies that published repeated data; (3) studies with levels of evidence < 2; (4) studies lacking the desired clinical outcomes.

### 2.2. Data Extraction Process

**Figure 1:**
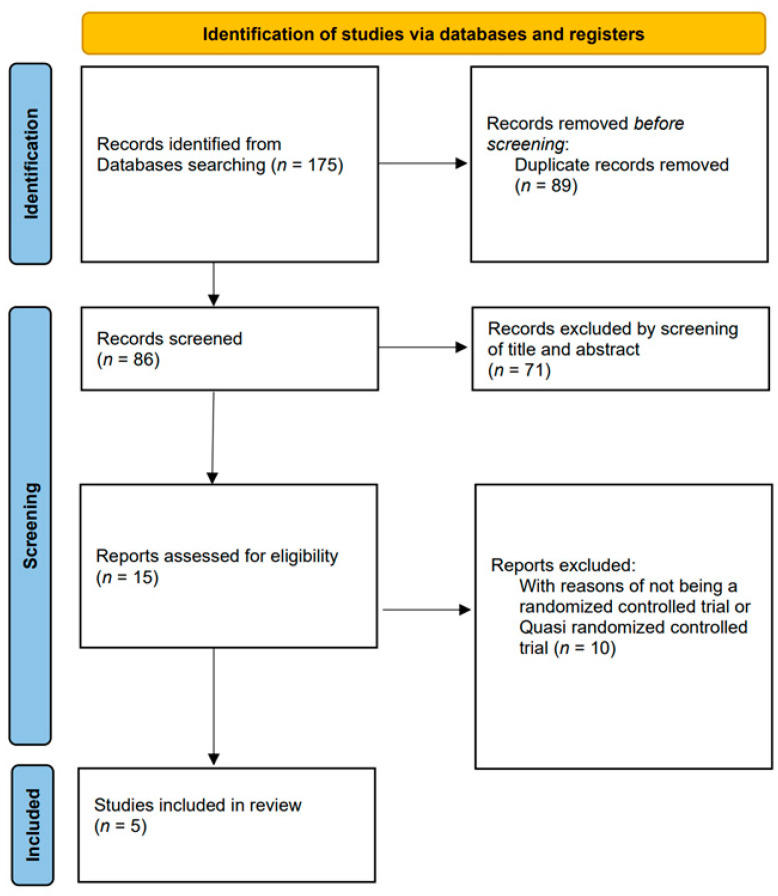
Flow chart for literature identification using the Preferred Reporting Items for Systematic Reviews and Meta-Analyses (PRISMA) guidelines.

The PRISMA flowchart summarizes the results of the literature search (Figure 1). Using the search terms described above, studies collected from each database were imported into Endnote X9. Two independent reviewers (H.G.S. and I.H.W.) screened the titles and abstracts of potentially eligible studies after eliminating duplicates. Thereafter, two authors (H.G.S. and J.J.P.) conducted a full-text review of the remaining potential studies, and the eligibility of each study was reassessed. Conflicts and disagreements were resolved by consensus with a third reviewer (J.J.P.). We then extracted data including the publication year, name of the first author, level of evidence of the study, demographic factors, number of patients, follow-up duration, complications, and clinical outcomes, such as the American Orthopedic Foot and Ankle Society (AOFAS) score, Ankle–Hindfoot Scale score, and Visual Analog Scale (VAS) score for pain.

### 2.3. Quality Assessment

For a high-quality meta-analysis, we included only randomized and quasi-randomized controlled trials. The methodologic quality of the included studies was assessed based on the Modified Coleman Methodology Score (MCMS) [30]. Each study was independently scored in duplicate by two authors. Two independent reviewers (I.H.W. and J.J.P.) assessed the risk of bias, while a third reviewer (H.G.S.) decided in cases where consensus was lacking [31].

We evaluated the randomized controlled trials (RCTs) using the Cochrane Risk of Bias tool (v2.0) [34] for assessing the risk of bias, which included following aspects: (1) random-sequence generation, (2) allocation concealment (selection bias), (3) blinding of participants and personnel (performance bias), (4) blinding of outcome assessment (detection bias), (5) incomplete outcome data (attrition bias), (6) selective reporting (reporting bias), and (7) other bias [34,35]. The selected studies were scored between 0 and 100, with an MCMS range of 85–100 considered ‘excellent’, 70–84 ‘good’, 55–69 ‘fair’, and <55 poor (Table 1).

### 2.4. Statistical Analysis

The meta-analysis was performed using RevMan 5.4 software. The heterogeneity was assessed using I2 statistics to measure the extent of inconsistency among the studies. A random-effects model was applied when homogeneity (I2 ≥ 50%) was observed. In contrast, when the I2 value was <50%, a fixed-effects model was used.

The standard mean difference (SMD) of the clinical outcomes was calculated to determine the treatment effect size and 95% confidence intervals (CIs) were obtained from the analysis. Statistical significance was set at *p* < 0.05.

## 3. Results

### 3.1. Study Selection and Characteristics of the Studies

A total of 175 potential studies were identified on PubMed (*n* = 36), Embase (*n* = 55), Web of Science (*n* = 76), and the Cochrane Library (*n* = 8). After removing duplicates, the titles and abstracts were reviewed, and 15 studies were considered appropriate for full-text screening. Nine studies were excluded after the full-text review. Finally, five studies were included in the meta-analysis. Of the five studies, four [6,29,30,31] were classified as randomized controlled trials, and the other study [28] was classified as a quasi-randomized controlled trial because complete randomization was not performed. Regarding the quality assessment, all five studies had a level of evidence above 2 and were of good or excellent quality in the MQOE. A total of 235 patients underwent an operation for OLT. There were 91, 95, and 49 patients in the MF, PRP + MF, and others (PRP + MF + other supplements augmentation) groups, respectively. In all included studies, the demographic characteristics such as the age, sex, etiology, location of the lesion, duration of symptoms, and body mass index were not different between the groups. Table 1 presents detailed characteristics of the studies.

### 3.2. Surgical Techniques of the Studies

Microfractures were performed consistently through all involved studies. In the ankle arthroscopy, the osteochondral lesions and necrotic or hypertrophic soft tissues were debrided. After confirming the location, size, and stability of the lesions, microfracture was plied with the lesions using different angled microfracture awls at optimal depth and intervals. Concerning the PRP application modalities, a single injection of 2–4 mL of PRP was performed 6 to 24 h after the operation in 3 studies [6,28,29], in one study [30] an intra-articular PRP injection was performed at the end of the arthroscopy during the operation, and in the other study [31] 4 consecutive weekly PRP injections were made after the operation. In terms of the other materials, BST-CarGel [28] was mixed with peripheral venous blood at a ratio of 3:1, then injected drop by drop with the help of an arthroscopic cannula at the end of the operation and 2 mL of hyaluronic acid (high-molecular-weight, cross-linked hyaluronic acid was injected 24 h after the operation) [29]. In the group undergoing mosaicplasty, using the Osteochondral Autograft Transfer System (OATS^®^, Acufex, Smith and Nephew, Andover, MA, USA) graft harvester, 2–3 osteochondral grafts were harvested from the ipsilateral knee joint. Samples appropriate in size and number for the contours of the talus defect were harvested from the ipsilateral knee joint. Graft tunnels appropriate for the height of osteochondral grafts were prepared using the apparatus in the OATS^®^ set [30].

### 3.3. AOFAS

**Figure 2:**
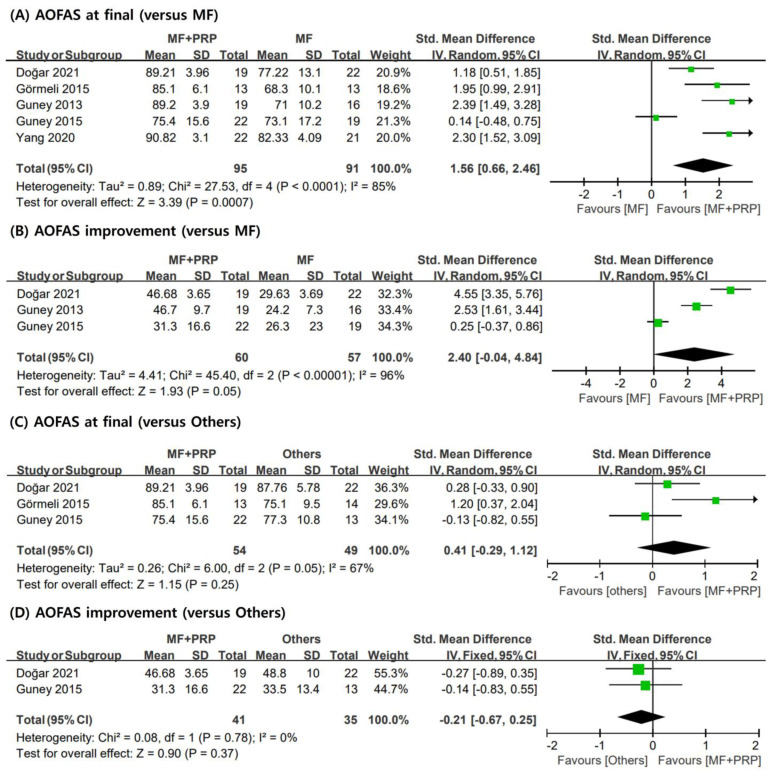
Meta-analysis results for AOFAS scores: (**A**) final AOFAS scores compared to MF only; (**B**) improvement of AOFAS scores compared to MF only; (**C**) final AOFAS scores compared to others group; (**D**) improvement of AOFAS scores compared to others group. AOFAS: American Orthopedic Foot and Ankle Society; MF: microfracture; PRP: plasma-rich platelet; SD: standard deviation.

In all studies [6,28,29,30,31], the AOFAS score was used to evaluate the functional outcomes for OLT. In this meta-analysis, the AOFAS scores at the final follow-up and the differences between pre- and post-operative points were analyzed. In the pooled analysis, no statistically significant difference was observed in the improvement of AOFAS scores (pooled SMD = 2.40; 95% CI = −0.04, 4.84; I2 = 96%) between the MF and PRP + MF groups. In contrast, the PRP + MF group had a higher AOFAS score at the final follow-up than that of the MF group (pooled SMD = 1.56; 95% CI = 0.66, 2.46; I2 = 85%). Additionally, no significant differences were observed in the MF + PRP and others groups (pooled SMD = 0.41; 95% CI = −0.29, 1.12; I2 = 67%). Figure 2 depicts the forest plots, SMD, 95% CI, and heterogeneity of the AOFAS scores.

### 3.4. VAS

**Figure 3:**
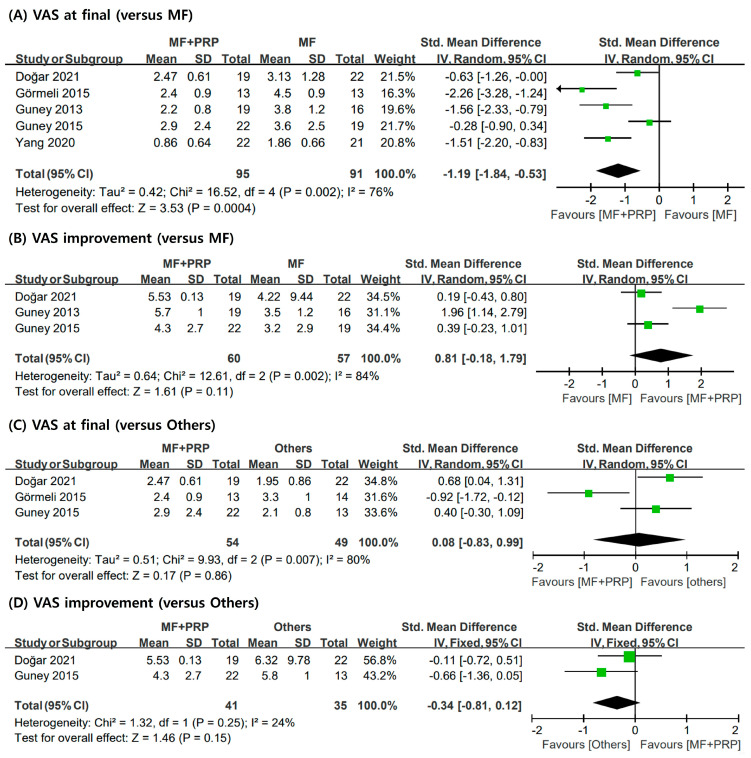
Meta-analysis results for VAS scores: (**A**) final VAS scores compared to MF only; (**B**) improvement of VAS scores compared to MF only; (**C**) final VAS scores compared to others group; (**D**) improvement of VAS scores compared to others group. VAS: Visual Analog Scale; MF: microfracture; PRP: plasma-rich platelet; SD: standard deviation.

Similarly, the VAS for pain was also used to evaluate the severity of pain in all included studies; the final VAS scores and changes in the scores were outcomes of interest. Three studies [6,28,30] reported the VAS scores before and after surgery, and these were used to evaluate changes in VAS. The random-effects model was used to analyze the final VAS scores and the change in the VAS scores. The post-operative VAS (SMD = −1.19; 95% CI = −1.84, −0.53; I2 = 76%) was significantly lower in the PRP + MF group than in the MF group. However, the improvement in the VAS scores were not significantly different between the two groups (SMD = 0.81; 95% CI = −0.18, 1.79; I2 = 84%). No remarkable difference between the MF + PRP and others groups was observed (SMD = 0.08; 95% CI = −0.83, 0.99; I2 = 80%) (Figure 3).

### 3.5. FAAM

**Figure 4:**
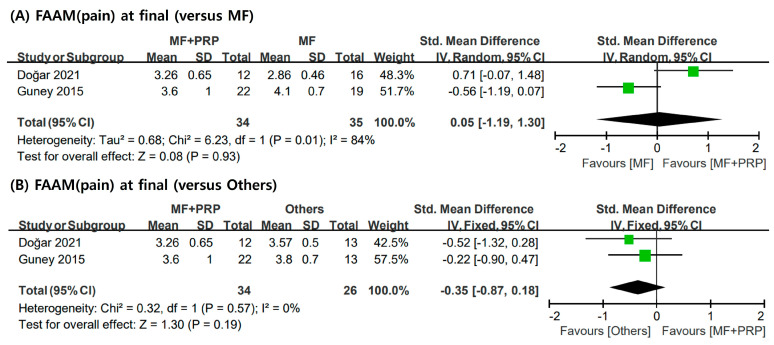
Meta-analysis results of the FAAM: (**A**) final FAAM scores compared to MF only; (**B**) final of FAAM scores compared to others group. FAAM: Foot and Ankle Ability Measure; MF: microfracture; PRP: plasma-rich platelet; SD: standard deviation.

The FAAM instrument evaluates the physical function of individuals with foot- and ankle-related impairments and consists of items such as overall pain, 15 min walking, and running. Figure 4 shows forest plots of pain-related items of the FAAM. Among the studies included in our meta-analysis, three studies [6,28,30] reported the FAAM score; however, only two studies [28,30] provided data suitable for analysis, and these were limited to overall pain. No significant difference was observed between the two groups in the overall pain as measured with the FAAM (pooled SMD = 0.05; 95% CI = −1.19, 1.30; I2 = 84%). Compared to the others group, the MF + PRP group showed no remarkable differences (pooled SMD = −0.35; 95% CI = −0.87, 0.18; I2 = 0%) (Figure 4)

### 3.6. Complications

The complications of PRP injections include the risks of joint infections, swelling, and post-injection pain. One study [29] did not report any post-operative complications. In contrast, the remaining four studies [6,28,30,31] reported no early or late complications in any patient.

### 3.7. Methodological Quality

**Figure 5:**
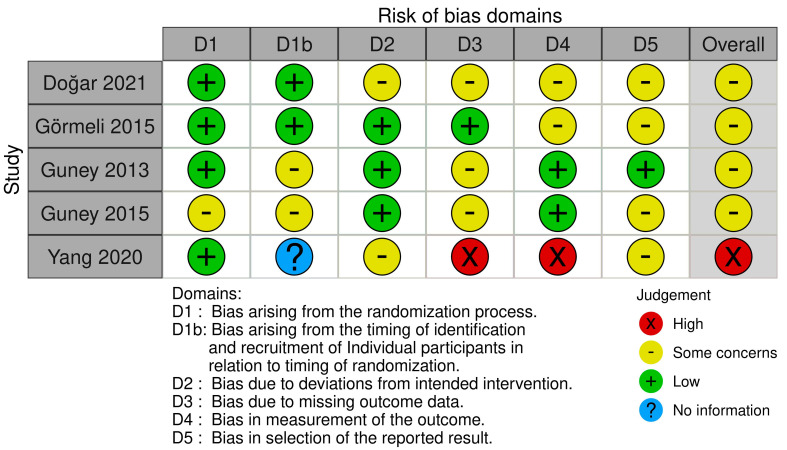
The methodological quality of the RCTs. Risk of bias summary: “+” means low risk; “?” means unclear risk; “−” means high risk.

The methodological quality of the RCTs is presented in Figure 5. All trials involved in this manuscript were RCTs.

### 3.8. Publication Bias

**Figure 6:**
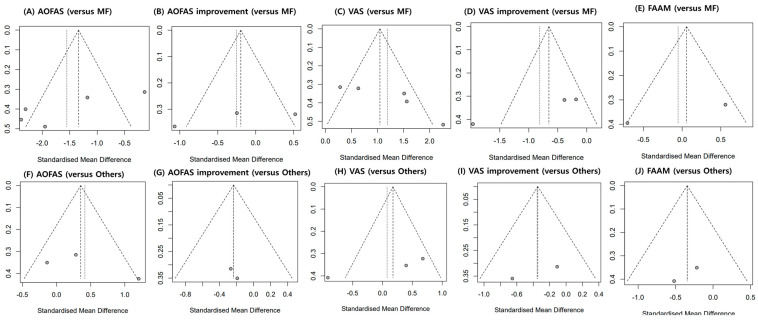
Graphic funnel plots of the included studies. AOFAS: American Orthopedic Foot and Ankle Society; VAS: Visual Analog Scale; FAAM: Foot and Ankle Ability Measure.

A funnel plot analysis was conducted to assess the publication bias (Figure 6). In addition, Egger’s test was performed for data reported in more than three studies. All factors had a *p*-value > 0.05 (AOFAS scores compared to MF only, *p* = 0.0697; improvement in AOFAS scores compared to MF only, *p* = 0.4057; VAS scores compared to MF only, *p* = 0.0622; improvement in VAS scores compared to MF only, *p* = 0.0651; AOFAS scores compared to other procedures, *p* = 0.4744; VAS scores compared to other procedures, *p* = 0.1368).

## 4. Discussion

The main important finding of this study is that PRP + MF improved the post-operative AOFAS and VAS scores compared with the MF only group. However, no significant pre- or post-operative changes were noted for either variable. No between-group differences were observed in the overall pain assessed using the FAAM tool. Furthermore, no comparable clinical improvements were observed over the others group.

Patients who have symptoms or progression in follow-up images require treatment. Surgical treatments can be considered if the progression was noted in an MRI, increasing cystic lesions or symptoms persist over 3 months, or conservative treatments have failed [36]. The surgical treatments include fixation, cartilage replacement therapy (autologous chondrocyte implantation, allograft transplantation, and osteochondral autograft transplantation), and bone marrow stimulation (microfracture, drilling, and abrasion arthroplasty. However, the proper selection of a surgical option remains a challenge for foot and ankle surgeons [37]. The surgical treatment strategies for OLT mainly involve bone marrow stimulation and cartilage replacement therapy [38]. MF, a bone marrow stimulation technique, has advantages such as swift recovery times, low morbidity rates, and cost-effectiveness, and is currently the first-line surgical treatment for small osteochondral lesions [39]. However, one study reported that the cartilage regeneration after MF surgery was neither homogenous nor intact, suggesting a weaker structure than hyaline cartilage [14]. In addition, the regenerated fibrous cartilage after MF is mechanically inferior to hyaline cartilage and is susceptible to deterioration over time [40,41]. Several studies have attempted to develop an intervention that can regenerate hyaline-like articular cartilage; however, the results of each study have limitations [14,42].

PRP contains growth factors with functions such as angiogenesis, collagen synthesis, and cell proliferation, and it can promote cartilage regeneration. PRP also enhances the repair of articular cartilage defects by increasing chondrocyte proliferation and inducing cartilaginous matrix formation [43,44]. In addition, PRP inhibits catabolic cytokines found in degenerative cartilage and suppresses inflammation by producing anti-inflammatory factors [45,46,47]. Various studies have demonstrated the effectiveness of PRP in various cartilage-related joint diseases of the hips and knees [48,49]. A study by Mei-Dan et al. [47] reported that PRP significantly improved the function and pain in patients with OLT. However, to our knowledge, only a few reviews [50,51] have compared PRP + MF and MF. Peng et al. [50] and Yausep et al. [51] pooled all PRP procedures in the same group, including simple injections of PRP and MF with PRP and joint distraction osteogenesis with PRP injection. In a previous meta-analysis by Boffa et al. [32], they reported the effects of PRP augmentation on the knee and ankle joints for cartilage lesions. However, we focused on OLT mainly and took a broader approach including more trials and comparing the clinical outcomes of PRP injections with MF over other augmentation materials.

Several studies have focused on PRP-augmented MF for OLT lesions [6,28,29,30,52]. However, the results reported in these studies are inconsistent. The AOFAS score is a widely used index to evaluate the functional outcomes of patients with foot and ankle injuries. Therefore, the AOFAS score was used as an important evaluation index in the studies included in this meta-analysis and other relevant studies. Fu et al. reported a positive relationship between the combination of a PRP injection with MF and AOFAS scores [52]. Moreover, Guney et al. [6] reported that patients in the PRP combined with MF group had better AOFAS scores and a markedly improved change (from baseline to follow-up) than the control group. In another study, the authors did not find any significant functional advantage of PRP + MF [30]. The results of our meta-analysis showed that the PRP combined with MF group had a superior final AOFAS score to the MF only group but no difference in the improvements of AOFAS scores from baseline was noted.

In this study, the VAS and FAAM scores were used to assess overall pain. Many previous studies have demonstrated that patients in the MF + PRP groups had lower VAS scores than the MF groups, which is consistent with the results of this study. However, no significant difference in the improvement of pain was noted between the two groups. The FAAM, like the AOFAS score, is an instrument that evaluates not only pain but also activities, such as walking and running. This score was used as an evaluation item in three studies [6,28,29,30] included in this study. Doğar et al. [28] described that the difference between and within the groups in post-operative FAAM scores for overall pain and VAS results were generally superior in the PRP + MF group compared with the MF group. In addition, Guney et al. [6] reported that the PRP group had a better overall pain level and 15 min walking distance than the controls at follow-up. In contrast, another study reported that the groups did not differ with regard to the FAAM scores for pain and FAAM scores for 15 min walking, which is consistent with the results of this meta-analysis [30].

Chitosan is a biodegradable and biocompatible polysaccharide that can adhere to tissues as a scaffold [28]. BST-CarGel (Primamal Life Sciences, Laval, QC, Canada) is a liquid form of chitosan solution that does not interfere in the bone canals after MF and tightens in the microfractured area [53]. It has been utilized for OLT to overcome the limitations of fibrocartilaginous tissue after MF [54]. Previous studies have already shown its successful results in hip and knee joints [55]. On the other hand, hyaluronic acid as a visco-supplementation [56,57], which refers to a synovial fluid replacement by intra-articular injection, has been widely used in ankle-specific diseases such as OLT and osteoarthritis. Several studies investigated these materials for OLT [28,29]. After Hangody et al. [58] introduced moscaicplasty, it showed clinically improved results, reporting 80–94% success rates. A good-to-excellent outcome was found in 92% of the 21 patients with OLT retrospectively [59]. From our present review, the differences between PRP + MF and MF + PRP + other substances were not significant. These results may be related to the limited data used to draw conclusions on these effects.

Side effects, such as infections, may lead to catastrophic results that require surgery and should be prevented. The most commonly reported complications of PRP injections include post-injection pain, swelling of the joint, dizziness, headaches, nausea, and sweating [60,61]. One case of cerebrovascular disease was reported by Paget et al. [62] that was considered unrelated to the intervention. No complications were reported in any of the included studies.

This meta-analysis has demonstrated the clinical outcomes of PRP + MF over MF or MF + PRP + other substances. Additionally, all included studies were high-quality studies with a level of evidence ≥ 2 and were randomized or quasi-randomized controlled trials. The quality of the studies evaluated using this methodological index was relatively high, with a minimum score of 73.

Our meta-analysis has several limitations. First, the relatively small number of patients may have biased the results and limited data were reported in the included studies. For high-quality analysis, we included only five papers in which the number of experimental and control groups was clearly described; therefore, a relatively small number of papers were included. Second, owing to the small number of studies, subgroup analyses on the dosage, frequency of PRP injection, or indications of the size of the affected lesion could not be performed. Secondly, due to the insufficient number of studies, all other studies using other adjuvant substances such as hyaluronic acid and BST-CarGel had to be combined into one group, “others”. However, the purpose of the present study was to obtain the comparative clinical outcomes of MF plus PRP rather than other treatment regimens. Individual study results were not taken into account, the conclusions may be interpreted. Finally, the results of the present meta-analysis provide no insights into the long-term clinical outcomes of PRP as an adjunct to MF in OLT. It is unclear whether PRP had any treatment benefit in addition to MF at five or more years of follow-up. Further research is required to elucidate the long-term effects of PR + MF.

## 5. Conclusions

In this meta-analysis, we compared the clinical outcomes of PRP + MF over MF only or PRP + MF + other supplements for OLT. The results of this analysis suggested that PRP + MF produced significantly superior outcomes compared with MF only in the final AOFAS and VAS scores and effective results over the others group. Therefore, the PRP + MF regimen led to enhanced pain alleviation and function recovery. The outcomes of the present meta-analysis can be a guide for the selection of surgical treatments for OLT. However, further studies are required to confirm their long-term efficacy.

## Figures and Tables

**Table 1:** Quality assessment and characteristics of the included studies.

Authors (Year)	Study Design (LOE)	MQOE	Intervention	Comparator(s)	Mean Age, Years	Follow Up (FU), Months	MF, *n*	MF + PRP, *n*	others, *n*	OLT size	Outcomes Recorded	Administration Modalities
Doğar et al. [28] (2021)	RCT (2)	83 (good)	Arthroscopic MF with PRP	Arthroscopic MF only/arthroscopic MF with BST-CarGel	MF: 33 ± 13.2 MF + PRP: 45.6 ± 11.8 MF + BST: 35.6 ± 12	Mean FU: MF: 24 ±15 MF + PRP: 30 ± 24 MF + BST: 25 ± 12.2 Range: -	22	19	22	Control: 1.2 cm^2^ MF: 1.14 cm^2^ MF + BST-CarGel: 1.5 cm^2^	AOFAS, FAAM, VAS, time to postoperative return to sport activity	Single injection of PRP (SmartPReP2 system) 24 h after the operation/BST-CarGel mixed with blood through arthroscopic cannula intraoperatively
Görmeli et al. [29] (2015)	RCT (1)	86 (excellent)	Arthroscopic MF with PRP	Arthroscopic MF with HA/arthroscopic MF with saline	MF + Saline: 40.3 ± 9.4 MF + HA: 39.7 ± 8.7 MF + PRP: 38.6 ± 9.1	15.3 Range: 11–25	13	13	14	PRP: 1.3 cm^2^ (0.5–1.4) Control: 1.2 cm^2^	AOFAS, VAS	Single injection of PRP (SmartPReP2 system) 6 and 24 h after the operation
Guney et al. [6] (2013)	RCT (2)	73 (good)	Arthroscopic MF with PRP	Arthroscopic MF only	MF: 42.8 ± 14.7 MF + PRP: 38.6 = 5 ± 12.7	Mean FU 16.2 Range 12–84	16	19	-	-	AOFAS, FAAM, VAS	Single injection of PRP (SmartPReP2 system [Harvest Autologous Hemobiologics]) 6 and 24 h after the operation
Guney et al. [30] (2015)	Quasi-RCT (2)	75 (good)	Arthroscopic MF with PRP	Arthroscopic MF only/mosaicplasy	MF: 33 ± 13.2 MF + PRP: 45.6 ± 11.8 Mosaicplasty: 35.6 ± 12	Mean FU: MF: 47.3 ± 16.9 MF + PRP: 40.4 ± 10.4 Mosaicplasty: 30.1 ± 13.1 Range: 12–24	19	22	13	PRP: <20 mm Control: <20 mm	AOFAS, FAAM, VAS	Single injection of PRP (SmartPReP2 system) 6 and 24 h after the operation
Yang et al. [31] (2020)	RCT (2)	78 (good)	Arthroscopic MF with PRP	Arthroscopic MF only	MF: 36.5 MF + PRP: 35.9	Mean FU: 15.6 Range: 12–18	21	22	-	0.93 ± 0.41 cm	AOFAS, VAS	Single injection of PRP (3 mL autologous PRP plus 10% CaCl_2_ 0.3 mL), 4 weekly injections after the operation

Abbreviations: RCT: randomized controlled trial; LOE: level of evidence; MQOE: methodologic quality of evidence; h: hour. Data are presented as means ± standard deviation. Dashes indicate information not reported.

## Data Availability

The data presented in this study are available on request from the corresponding author.
